# Prognostic Impact of IPSS-R and Chromosomal Translocations in 751 Korean Patients with Primary Myelodysplastic Syndrome

**DOI:** 10.1371/journal.pone.0166245

**Published:** 2016-11-08

**Authors:** Koung Jin Suh, June-Won Cheong, Inho Kim, Hyeoung-Joon Kim, Dong-Yeop Shin, Youngil Koh, Sung-Soo Yoon, Yoo Hong Min, Jae-Sook Ahn, Yeo-Kyeoung Kim, Yun-Gyoo Lee, Jeong-Ok Lee, Soo-Mee Bang, Yeung-Chul Mun, Chu-Myoung Seong, Yong Park, Byung-Soo Kim, Junshik Hong, Jinny Park, Jae Hoon Lee, Sung-Yong Kim, Hong Ghi Lee

**Affiliations:** 1 Department of Internal Medicine, Seoul National University Hospital, Cancer Research Institute, Seoul National University College of Medicine, Seoul, Korea; 2 Department of Internal Medicine, Yonsei University Severance Hospital, Seoul, Korea; 3 Department of Internal Medicine, Chonnam National University Hwasun Hospital, Hwasun, Chonnam, Korea; 4 Department of Internal Medicine, Kangbuk Samsung Hospital, Seoul, Korea; 5 Department of Internal Medicine, Seoul National University Bundang Hospital, Seongnam, Gyeonggi, Korea; 6 Department of Internal Medicine, Ewha Womans University Mokdong Hospital, Seoul, Korea; 7 Departmenet of Internal Medicine, Korea University Anam Hospital, Seoul, Korea; 8 Department of Internal Medicine, Gachon University Gil Medical Center, Incheon, Korea; 9 Department of Internal Medicine, Konkuk University School of Medicine, Seoul, Korea; Hospital Universitario de Salamanca, SPAIN

## Abstract

Chromosomal translocations are rare in myelodysplastic syndrome (MDS) and their impact on overall survival (OS) and response to hypomethylating agents (HMA) is unknown. The prognostic impact of the revised International Prognostic Scoring System (IPSS-R) and for chromosomal translocations was assessed in 751 patients from the Korea MDS Registry. IPSS-R effectively discriminated patients according to leukaemia evolution risk and OS. We identified 40 patients (5.3%) carrying translocations, 30 (75%) of whom also fulfilled complex karyotype criteria. Translocation presence was associated with a shorter OS (median, 12.0 versus 79.7 months, *P* < 0.01). Multivariate analysis demonstrated that translocations (hazard ratio [HR] 1.64 [1.06–2.63]; *P* = 0.03) as well as age, sex, IPSS-R, and CK were independent predictors of OS. In the IPSS-R high and very high risk subgroup (n = 260), translocations remained independently associated with OS (HR 1.68 [1.06–2.69], *P* = 0.03) whereas HMA treatment was not associated with improved survival (median OS, 20.9 versus 21.2 months, *P* = 0.43). However, translocation carriers exhibited enhanced survival following HMA treatment (median 2.1 versus 12.4 months, *P* = 0.03). Our data suggest that chromosomal translocation is an independent predictor of adverse outcome and has an additional prognostic value in discriminating patients with MDS having higher risk IPSS-R who could benefit from HMA treatment.

## Introduction

Myelodysplastic syndromes (MDS) are a heterogeneous group of clonal myeloid disorders characterized by ineffective haematopoiesis resulting in bone marrow (BM) failure and increased risk of transformation to acute myeloid leukaemia (AML) [1]. Chromosomal translocations are rare in MDS, whereas other chromosomal abnormalities such as losses and gains of genetic material are detected in half of all patients with MDS. The International Prognostic Scoring System (IPSS) [2] and revised IPSS (IPSS-R) [3] comprise the most accepted prognostic scoring systems incorporating 3 and 5 cytogenetic prognostic subgroups, respectively, yet chromosomal translocations other than t(3q) are not considered in the cytogenetic classification. Owing to recent advances in technologies such as whole genome sequencing, recurrent mutations in splicing factor (e.g., *SF3B1* and *U2AF1*) and epigenetic regulator (e.g., *TET2*, *ASXL2*, *DNMT3A*, *EZH2*, and *IDH1/2*) genes have been identified as the predominant molecular mechanism underlying the pathogenesis of MDS [4–6]. However, since the identification and reporting of recurrent chromosomal translocations are essential to provide insights into the mechanisms of pathogenesis and identify genes involved in the pathogenesis of MDS, the role of cytogenetics including the study of genomic structure and function will remain important. Novel and recurrent translocations have been reported in a large series of patients [7,8]; however, only a few studies have addressed the prognostic impact of chromosomal translocations [9–11].

Accordingly, the aims of this study were: (1) to validate the prognostic power of IPSS-R in a large cohort of adults with primary MDS and (2) to assess the incidence, characteristics, and potential prognostic impact of chromosomal translocation in this cohort.

## Patients and Methods

### Study population

The Korea MDS registry which is sponsored by the Korean Society of Haematology holds data from major centres in Korea that treated patients with MDS between January 2004 and December 2011. We conducted a retrospective study of these data, resulting in the enrolment of a total of 751 consecutive patients for whom the complete data set needed to calculate the IPSS or IPSS-R score. We excluded patients with secondary MDS, chronic myelomonocytic leukemia (CMML), and patients with AML and a BM blast count of 20–30% who were classified as having refractory anaemia with excess blast (RAEB)-t according to the French-American-British classification [12]. Patients were then reclassified according to the 2008 WHO criteria [13]. The individual review boards of all participating institutions approved this study, and it was conducted in accordance with the Declaration of Helsinki.

### Cytogenetic analyses

Cytogenetics was evaluated using BM cells with standard banding techniques and the cytogenetic abnormalities were classified according to the International System for Human Cytogenetic Nomenclature criteria [14]. At least 20 metaphases per sample were analysed when possible and the cytogenetic reports were centrally reviewed. Aberrations were counted following the International Working Group on MDS Cytogenetics (IWGMC) consensus guidelines [15]. Patients with 3 or more choromosmal abnormalities were defined having complex karyotype (CK). Monosomal karyotype (MK) was defined as the presence of at least 2 autosomal monosomies or 1 single autosomal monosomy in combination with structural abnormalities. The Atlas of Genetics and Cytogenetics in Oncology and Haematology [http://atlasgeneticsoncology.org] was reviewed to determine whether the translocations in our study population had been previously reported.

### Statistical analysis

Patient characteristics between groups were compared using the *χ*2-test, Fisher’s exact test, or Mann Whitney U-test as appropriate. Overall survival (OS) was defined as the duration of time between the date of first diagnosis and the date of last follow-up or death from any cause. The period of leukaemia-free survival (LFS) was defined as the duration of time between the date of diagnosis to the date of AML evolution or death from any cause, whichever came first. OS and LFS were estimated using the Kaplan-Meier method and the values were compared by the log-rank test. Univariate Cox proportional-hazard regression (PHR) analyses were performed to evaluate the prognostic values of each variable and variables found to be significant on univariate analysis were introduced into a multi-variable Cox PHR model for LFS and OS. All tests were two-sided, and a *P* value of less than 0.05 indicated a statistically significant difference. All analyses were performed using SPSS Version 22.0 (SPSS; Chicago, IL, USA) and GraphPad Prism 5 (GraphPad Software, Inc., La Jolla, CA, USA) on data collected through December 2015.

## Results

### Patient characteristics

The clinical characteristics of 751 patients are shown in Table 1. The median age of the patients was 65 years, and 457 (61.9%) were male. The median follow-up time was 98.5 months (range, 38.1–246.6). The most common WHO subtype was refractory cytopaenia with multilineage dysplasia (29.7%), followed by RAEB-1 (19.8%), RAEB-2 (18.4%), refractory cytopaenia with unilineage dysplasia (15.4%), and MDS-unclassifiable (11.9%). More than half of patients received disease-modifying treatment; 381 (50.7%) received hypomethylating agents (HMAs), and 83 (11.1%) received haematopoietic stem cell transplantation (HSCT).

**Table 1:** Baseline Characteristics of 751 patients with MDS.

Variables		n	Translocation (n = 40)	No translocation (n = 711)	*P-*value
Age (years)	<60	286	10 (25.0)	276 (38.8)	0.08
	≥60	465	30 (75.0)	435 (61.2)	
Gender	Male	457	23 (57.5)	434 (61.0)	0.66
	Female	294	17 (42.5)	277 (39.0)	
ANC (x 109/L) (median, range)			0.86 (0.67–6.99)	1.20 (0–52.99)	0.36
Hb (g/dL) (median, range)			8.1 (4.4–14.5)	8.5 (2.8–15.9)	0.30
Platelets (x 1012/L) (median, range)			72 (6–288)	76 (1–4120)	0.33
BM blasts (%) (median, range)			5.4 (0–19.8)	2.5 (0–20)	<0.01
Diagnosis	RARS	28	0	28 (4.0)	<0.01
	RCUD	115	2 (5.0)	113 (16.0)	
	RCMD	223	9 (22.5)	214 (30.3)	
	RAEB-1	149	9 (22.5)	140 (19.8)	
	RAEB-2	138	18 (45.0)	120 (17.0)	
	MDS-U	89	2 (5.0)	87 (12.3)	
	Del5q	5	0	5 (0.7)	
	Unknown	4	0	4 (0.6)	
IPSS	Low	140	0	140 (19.7)	<0.01
	Int-1	419	7 (17.5)	412 (57.9)	
	Int-2	150	20 (50)	130(18.3)	
	High	42	13 (32.5)	29 (4.1)	
IPSS-R	Very low	51	0	51 (7.2)	<0.01
	Low	221	1 (2.5)	220 (30.9)	
	Intermediate	219	4 (10.0)	215 (30.2)	
	High	152	9 (22.5)	143 (20.1)	
	Very high	108	26 (65.0)	82 (11.5)	
HMA	Yes	381	27 (67.5)	354 (49.8)	0.03
	No	370	13 (32.5)	347 (50.2)	
HSCT	Yes	83	3 (7.5)	80 (11.3)	0.46
	No	668	37 (92.5)	631 (88.7)	

Abbreviations: MDS, myelodysplastic syndrome; ANC, absolute neutrophil count; Hb, hemoglobin; BM, bone marrow; WHO, World Health Organization; RARS, refractory anemia with ringed sideroblasts; RCUD, refractory cytopenia with unilineage dysplasia; RCMD, refractory cytopenia with multilineage dysplasia; RAEB, refractory anemia with excess blast; MDS-U, MDS unclassified; IPSS, International Prognostic Scoring System; IPSS-R, Revised IPSS; HMA, hypomethylating agent; HSCT, hematopoietic stem cell transplantation.

### Analysis of IPSS and IPSS-R

**Fig 1:**
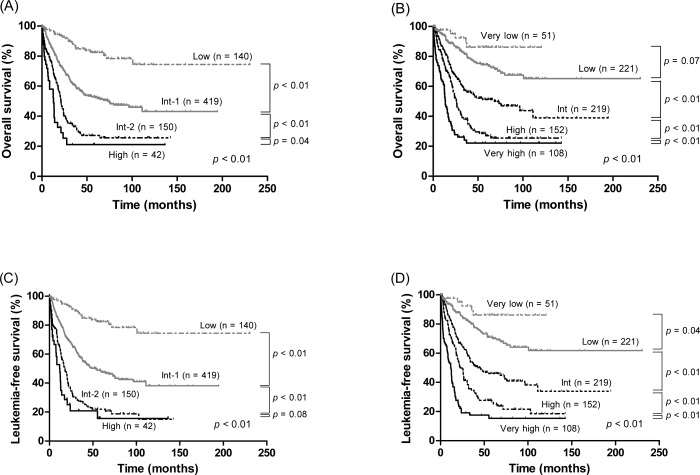
Kaplan-Meier survival curves of overall survival (A and B) and leukemia-free survival (C and D) in 751 patients with primary MDS stratified by IPSS and IPSS-R.

We calculated the IPSS and IPSS-R scores at diagnosis. According to the IPSS classification, 140 (18.6%) patients were considered to be low-risk, 419 (55.8%) intermediate-1 risk, 150 (20.0%) intermediate-2 risk, and 42 (5.6%) high-risk. The OS among these 4 groups were significantly different (not reached [NR], 73.0, 21.0, and 12.9 months for IPSS low, intermediate-1, intermediate-2, and high risk, respectively; *P* < 0.01) (Fig 1A). There was also a statistically significant difference in LFS among these 4 groups (*P* < 0.01, Fig 1C). However, we could not identify an intergroup difference in LFS between the intermediate-2 and high risk groups (*P* = 0.08). According to the IPSS-R, 51 patients (6.8%) were considered to be very low-risk, 221 (29.4%) low-risk, 219 (29.2) intermediate-risk, 152 (20.2%) high-risk, and 108 (14.4%) very high-risk. For these groups, the median survivals were NR, NR, 68.2, 25.9, and 13.5 months, respectively (*P* < 0.01) (Fig 1B). However, we could not identify an intergroup difference in OS between the very low and low risk groups (*P* = 0.07). IPSS-R was able to stratify patients with respect to LFS (*P* < 0.01, Fig 1D).

### Chromosomal translocation in patients with MDS

A total of 291 patients (38.7%) demonstrated an abnormal karyotype, of whom 40 had chromosomal translocations, representing 5.3% of all patients and 13.7% of patients with abnormal karyotype. Among these, 46 translocations involving 72 breakpoints were identified including balanced translocations in 13 (28.3%) and unbalanced in 33 (71.7%). CK and MK were found in 91 and 73 patients representing 31.3% and 25.1% of patients with an abnormal karyotype, respectively. Translocations were found as a part of CK in 30/46 (65.2%) cases and as a part of MK in 21/46 (45.7%). Table 2 illustrates the details of the patients with chromosomal translocations including diagnosis, treatment, and duration of survival. All chromosomes except for chromosome 18 and the Y chromosome participated in the translocations. Frequently involved chromosomes were chromosome 7 (n = 13); ch1 (n = 12); ch5 (n = 9); ch12 (n = 6); ch6, ch11, and ch14 (n = 5); ch10 and ch20 (n = 4); and ch16, ch17, and X (n = 3) in decreasing order. To the best of our knowledge, 43 (93.5%) of the 46 translocations have not been previously described in MDS, nor in AML or CMML. All translocations involved breakpoints recurrently involved in translocations with other partners in MDS, CMML, and AML. Some of the breakpoints (29/72, 40.3%) included genes related to MDS or AML (Table 2). Patients with chromosomal translocation exhibited worse prognostic baseline characteristics compared with those without translocation (Table 1). Patients with translocation also had a higher percentage of BM blasts and included a significantly larger proportion of RAEB-1 and RAEB-2. The raw number of chromosomal aberrations were higher in the patients with translocation compared with those without in all patients (median number of aberration 1.0 [range, 0–16.0] versus 5.5 [1.0–22.0], *P* < 0.01); identical results were obtained when the analysis was restricted to the patients with any chromosomal aberration (2.0 [1.0–16.0] versus 5.5 [1.0–22.0], *P* < 0.01).

**Table 2:** Balanced and Unbalanced Translocations in MDS patients.

No.	Age/ Sex	Translocation	Karyotype	Diagnosis	Translocation reported	Genes (breakpoints)	IPSS-R	Tx	AL	OS (mo)
1	64/M	der(7)t(1;7)(q21;q11.2)	CK	RAEB-2	1q21^b^^,^^c^ / 7q11^a^	HIP1 (7q11)	VH	HMA	No	0.6 (Died)
2	67/M	der(20)t(12;20)(q13;q13.3)	CK, MK	RCMD	12q13^b^^,^^c^ / 20q13^b^^,^^c^	NCOA3 (20q13)	VH	HMA	No	1.5 (Died)
3	65/M	der(3)t(3;7)(p14;p13)	CK, MK	RAEB-1	3p14^a^ / 7p13^a^^,^^c^	-	VH	HMA	No	8.2 (Died)
4	86/F	t(4;16;12)(q25;p13.3;q13)	AA	RAEB-1	4q25^b^ / 16p13^b^ / 12q13^b^^,^^c^	CREBBP, GLIS2, MYH11 (16p13)	I	BSC	No	58.7 (Alive)
5	42/F	der(5)t(5;11)(q22;q13)	CK	RAEB-1	5q22^b^^,^^c^ / 11q13^b^	MACROD1, NUMA1 (11q13)	VH	HMA	Yes	13.3 (Died)
6	75/M	der(14;20)t(14;20)(q10;p10)	CK, MK	RAEB-1	14q10^b^ / 20p10^a^	-	VH	BSC	No	2.1 (Died)
7	60/F	der(1)t(1;7)(q10;p10)	MK	MDS-U	1q10^b^ / 7p10^b^^,^^c^	-	I	HSCT	No	20.3 (Died)
8	64/M	t(3;?)(q21;?)	CK, MK	RAEB-2	3q21^b^	GATA2, RPN1 (3q21)	VH	HMA	No	1.0 (Died)
9	78/F	der(12)t(12;14)(p13;q11.2)	CK, MK	RCMD	12p13^b^ / 14q11^a^^,^^c^	ETV6, KDM5A, ZNF384 (12p13)	VH	BSC	No	0.2 (Died)
10	74/F	der(11)t(11;11)(q23;p15)	CK	RAEB-2	11p15^b^ / 11q23^b^	NUP98 (11p15), MLL (11q23)	VH	HMA	Yes	12.6 (Died)
11	66/M	der(5)t(X;5)(q22;q13)	CK	RAEB-1	Xq22^a^ / 5q13^b^^,^^c^	-	VH	BSC	No	0.5 (Died)
12	83/M	der(5)t(5;7)(q22;p13)	CK, MK	RCMD	5q2^b^ / 7p13^a^^,^^c^	-	VH	HMA	No	12.2 (Loss)
13	65/M	t(7;10)(q10;p10)	CK, MK	RAEB-2	7q10^b^ / 10p10^a^	-	VH	HMA	No	14.1 (Died)
14	77/F	der(7)t(1;7)(p22;p10), der(17)t(8;17)(q13;p12)	CK, MK	RAEB-2	1p22^b^^,^^c^ / 7p10^b^^,^^c^ / 8q13^a^ / 17p12^b^	CLCA2 (1p22), NCOA2 (8q13)	VH	HMA	Yes	12.5 (Loss)
15	68/M	t(17;21)(q21;q22)	AA	RCMD	Acute leukemia		H	HMA	No	31.8 (Died)
16	76/M	der(16)t(7;16)(p15;p13.1), t(1;6)(p31;q21), t(12;15)(q13;q11.2)	CK, MK	RAEB-2	7p15^b^ / 16p13^b^ / 1p31^a^ / 6q21^b^ / 12q13^b^^,^^c^ / 15q11^a^^,^^c^	HOXA9 (7p15), FOXO3 (6q21)	VH	HMA	Yes	13.5 (Died)
17	72/M	der(22)t(22;?)(q10;?)	CK, MK	RCMD	22q10^a^	-	VH	BSC	No	2.4 (Died)
18	60/F	der(16)t(16;17)(q13;q11.2)	CK, MK	RAEB-2	16q13^b^ / 17q11^a^	KSR1, WSB1, MYO18A, GOSR1, ZNF207, NF1 (17q11)	VH	HMA	Yes	23.4 (Died)
19	31/F	der(8)t(8;20)(q10;q12)	CK	RCUD	8q10^b^ / 20q12^b^	TOP1 (20q12)	H	BSC	No	12.9 (Loss)
20	42/M	der(6)t(1;6)(q21;p23)	AA	RAEB-1	1q21^b^^,^^c^ / 6p23^b^^,^^c^	-	H	HMA	No	2.3 (Died)
21	60/M	der(5)t(5;15)(q13;q11.2)	CK, MK	RCMD	5q13^b^^,^^c^ / 15q11^a^^,^^c^	-	H	HMA	No	9.9 (Died)
22	52/M	der(7)t(1;7)(q12q36)	AA	RAEB-2	7q36^b^	EZH2, MNX1 (7q36)	VH	HMA	Yes	6.5 (Died)
23	71/M	t(1;7)(p22;q32)	CK, MK	RAEB-1	1p22^b^^,^^c^ / 7q32^b^	-	VH	HMA	No	1.1 (Died)
24	60/M	der(2)t(2;10)(p?25;q?22), der(5)t(5;14)(q22;q11.2), der(6)t(6;10)(p23;q22)	CK, MK	RCMD	2p25^a^ / 10q22^b^^,^^c^ / 5q22^b^^,^^c^ / 14q11^a^^,^^c^ / 6p23^b^^,^^c^	KAT6B, KCNMA1 (10q22)	VH	HMA	No	1.5 (Died)
25	56/M	t(5;7)(q13;q22)	CK	RAEB-2	5q13^b^^,^^c^ / 7q22^b^	ZNF789, ZNF394 (7q22)	H	HMA	No	57.2 (Loss)
26	67/F	der(17)t(12;17)(q12;p11.2)	CK, MK	RAEB-2	12q12^a^ / 17p11^b^	CPNE8 (12q12), SPECC1 (17p11)	VH	HMA	No	33.7 (Loss)
27	34/F	der(7)t(6;7)(p21;p22)	CK, MK	RAEB-2	AML		VH	HSCT	Yes	13.4 (Died)
28	63/F	t(1;14)(p36.1;q24)	CK	RAEB-2	1p36^b^ /14q24^a^	CDK11B, PRDM16, MTOR (1p36)	VH	HMA	No	12.4 (Died)
29	57/F	der(16)t(1;16)(q21;q22)	A	MDS-U	1q21^b^^,^^c^ /16q22^b^	CBFB, NFATC3, PLA2G15 (16q22)	I	BSC	No	96.7 (Alive)
30	77/F	der(12)t(7;12)(p11.2;p11.2)	CK, MK	RAEB-2	7p11^b^ / 12p11^b^	FGFR1OP2 (12p11)	VH	HMA	No	5.0 (Loss)
31	56/M	der(6)t(6;13)(p12;q22)	CK, MK	RAEB-2	6p12^a^ / 13q22^b^	CLN5, FBXL3 (13q12)	VH	HMA	Yes	6.8 (Died)
32	35/F	t(1:19)(q21:q13)	CK	RCMD	1q21^b^^,^^c^ /19q13^b^	CEBPA (19q13)	H	BSC	No	0.9 (Died)
33	64/F	t(9;22)(q34;q11.2)	CK	RAEB-2	AML, RAEB-2		VH	HMA	No	7.2 (Died)
34	65/M	der(1)t(1;10)(p13;q24)	A	RAEB-1	1p13^b^ / 10q24^a^	NRAS, RBM15, DDX20 (1p13), GOT1, NT5C2 (10q24)	H	HMA	No	21.0 (Died)
35	65/F	del(20)t(1;20)(q11;q13.3)	A	RCUD	1q11^b^ / 20q13^b^^,^^c^	NCOA3 (20q13)	I	BSC	No	7.1 (Died)
36	67/M	del(13)t(5;13)(q21;q14),der(7)t(5;7)(p14;p13)	CK, MK	RAEB-2	5q21^b^ / 13q1^b^, 5p14^a^ / 7p13^a^^,^^c^	-	H	HMA	No	12.0 (Died)
37	76/F	t(X;11)(q13;q25)	CK, MK	RAEB-1	Xq13^b^/ 11q25^b^	FOXO4 (Xq13)	VH	HMA	Yes	4.0 (Died)
38	70/M	t(4;14)(q35;q22)	A	RCMD	4q35^a^ / 14q22^b^	-	L	BSC	No	88.5 (Alive)
39	79/M	t(11;21)(p13;q21)	CK, MK	RAEB-2	21q22^b^	RUNX1 (21q22)	VH	BSC	No	3.2 (Died)
40	58/M	t(x:5)(q24;p15.3)	AA	RAEB-2	Xq24^a^ / 5p15^a^	SEPT6 (Xq24)	H	HMA, HSCT	Yes	20.7 (Died)

Abbreviations: CK, complex karyotype; MK, monosomal karyotype; AA, associated with another chromosomal abnormality; A, isolated chromosomal abnormality; RCUD, refractory cytopenia with unilineage dysplasia; RCMD, refractory cytopenia with multilineage dysplasia; RAEB refractory anemia with excess blasts; MDS-U, MDS unclassified;.IPSS-R, revised International Prognostic Scoring System; Tx, treatment; HMA, hypomethylating agent; BSC, best supportive care; HSCT, hematopoietic stem cell transplantation; AL, evaluation to acute leukemia; OS, overall survival.

^a^Breakpoints reported in AML.

^b^Breakpoints reported in both AML and MDS.

^c^Breakpoints reported more than 2 times in this study population.

### Effect of chromosomal translocation on outcome

**Fig 2:**
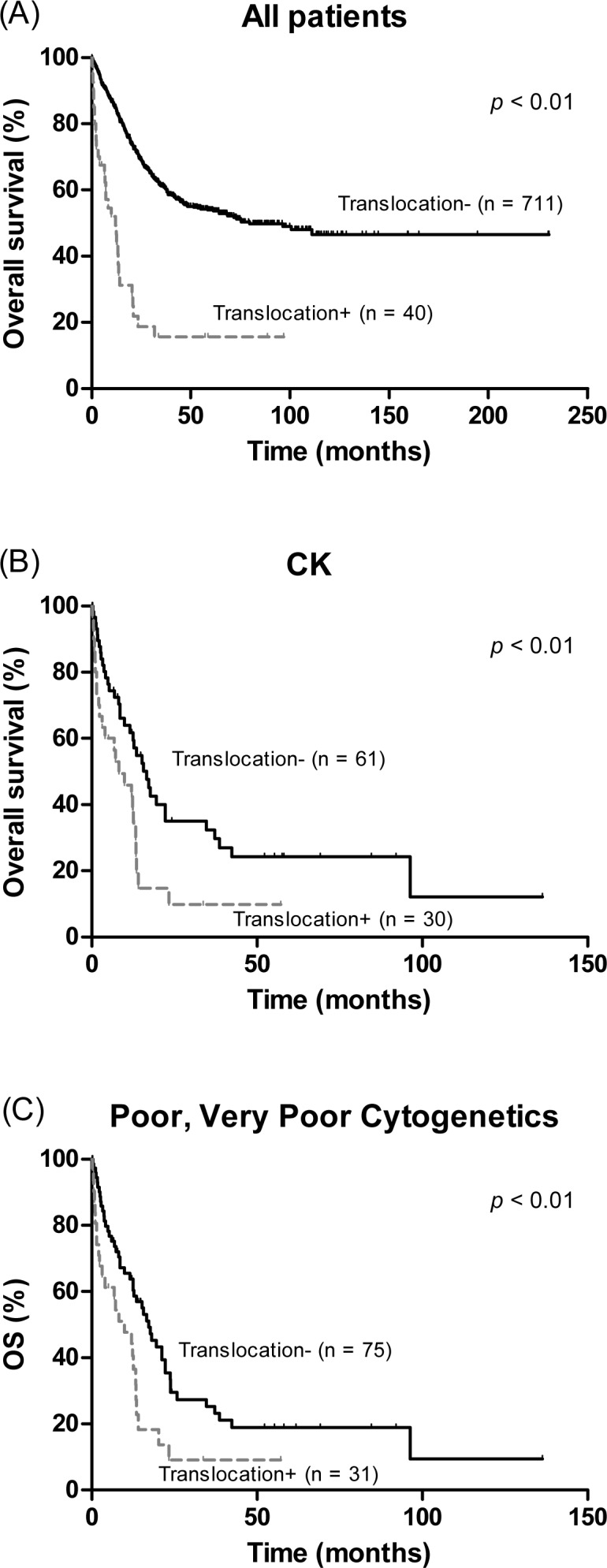
Overall survival according to presence of chromosomal translocation in (A) the whole population, and (B) patients with complex karyotype (CK), and (C) patients with IPSS-R poor and very poor cytogenetics.

Patients with chromosomal translocation had markedly inferior OS in comparison with the rest of the patient cohort (median OS, 12.0 versus 79.7 months, *P* < 0.01) (Fig 2A). Patients with MK or CK also exhibited worse survival than those without (median OS, 12.8 versus 100.6 months, *P* < 0.01 and 12.8 versus 111.0 months, *P* < 0.01, respectively). Among patients with CK, the presence of MK did not have an impact on either OS or LFS (median OS, 9.9 months for MK versus 17.3 months for non-MK, *P* = 0.21 and median LFS 8.5 months for MK versus 14.1 for non-MK, *P* = 0.23, respectively) (S1 Fig). In contrast, presence of a chromosomal translocation was sufficient to stratify the CK group; patients with translocation had distinctly poorer OS and LFS than those without (median 8.2 versus 16.6 months, *P* < 0.01 and median 7.2 versus 12.8 months, *P* < 0.01, respectively) (Fig 2B). Among 106 patients with very poor and poor karyotype, 31 carried translocations. The presence of a translocation also adversely affected survival (Fig 2C).

Of the 40 patients with translocations, 13 had translocation involving chromosome 7. Patients with chromosome 7 translocation had similar OS and LFS compared with the patients with translocation involving other chromosomes (*P* = 0.72 and *P* = 0.63, respectively). Clinical outcome of patients with chromosome 7 translocation was also compared with those of patients with monosmy 7. Seven patients had translocation involving chromosome 7, twenty-four patients had monosomy 7, and six patients had both abnormalities. OS did not differ significantly according to the type of abnormality involving chromosome 7 (median OS 12.0 months for chromosome 7 translocation *vs*. 8.5 months for monosomy 7 *vs*. 14.1 months for both abnormalities, *P* = 0.62).

Of the 40 patients with translocations, 7 had translocation involving 5q, which has been suggested as a poor prognostic cytogenetics [16]. After excluding 7 patients with t(5q), patients with translocation still had markedly inferior OS and LFS in comparison with the rest of the patient cohort (median OS, 12.4 versus 75.3 months, *P* < 0.01; median LFS, 7.4 versus 55.3 months, *P* < 0.01) (S2 Fig).

Among the entire cohort of 751 patients, chromosomal translocation remained a significant variable associated with shorter OS in multivariate analysis (HR 1.64, 95% CI 1.06–2.54, *P* = 0.03). Other variables that retained statistical significance for OS were: age > 60 years (HR 1.61, 95% CI 1.21–2.15, *P* < 0.01), male sex (HR 1.42, 95% CI 1.10–1.84, *P* < 0.01), CK (HR 1.75, 95% CI 1.17–2.63, *P* < 0.01), and higher IPSS-R risk group (*P* < 0.01) (Table 3). Chromosomal translocation was independently associated with LFS as well with the same variables as entered in the regression model (HR 1.56 [1.03–2.43], *P* = 0.04). Chromosomal translocation remained a significant variable associated with shorter OS (HR 1.89, 95% CI 1.21–2.95, *P* < 0.01) and LFS (HR 1.80, 95% CI 1.17–2.77, *P* < 0.01) even after excluding 7 patients with t(5q) (S1 Table).

**Table 3:** Multivariate analyses for OS and LFS.

Variables	HR for OS (95% CI)	*p*-value	HR for LFS (95% CI)	*P*-value
Age				
< 60	1		1	
≥ 60	1.61 (1.21–2.15)	< 0.01	2.07 (1.61–2.67)	<0.01
Sex				
Female	1			
Male	1.42 (1.10–1.84)	< 0.01		NSS
Translocation				
No	1		1	
Yes	1.64 (1.06–2.54)	0.03	1.58 (1.03–2.43)	0.04
CK				
No	1		1	
Yes	1.75 (1.17–2.63)	< 0.01	1.70 (1.10–2.63)	0.02
IPSS-R				
Very Low	1		1	
Low	2.82 (1.01–7.86)	0.05	2.66 (1.06–6.68)	0.04
Intermediate	6.20 (2.27–16.95)	< 0.01	5.11 (2.05–12.72)	<0.01
High	10.78 (3.93–29.57)	< 0.01	6.26 (2.43–16.12)	<0.01
Very High	12.31 (4.33–34.96)	< 0.01	8.92 (3.32–23.98)	<0.01

Abbreviations: HR, hazard ratio; CI, confidence interval; IPSS-R, Revised International Prognostic Scoring System; OS, overall survival; CK, complex karyotype; NSS, not statistically significant.

When the analysis was restricted to patients with chromosomal abnormalities (n = 271), translocation was a significant predictor of poor OS and LFS (HR for OS, HR 1.856, 95% CI 1.198–2.876, *P* < 0.01; HR for LFS, 1.733, 95% CI 1.132–2.653, *P* = 0.01).

### Analysis of the prognostic factors of patients with higher risk according to the IPSS-R

**Fig 3:**
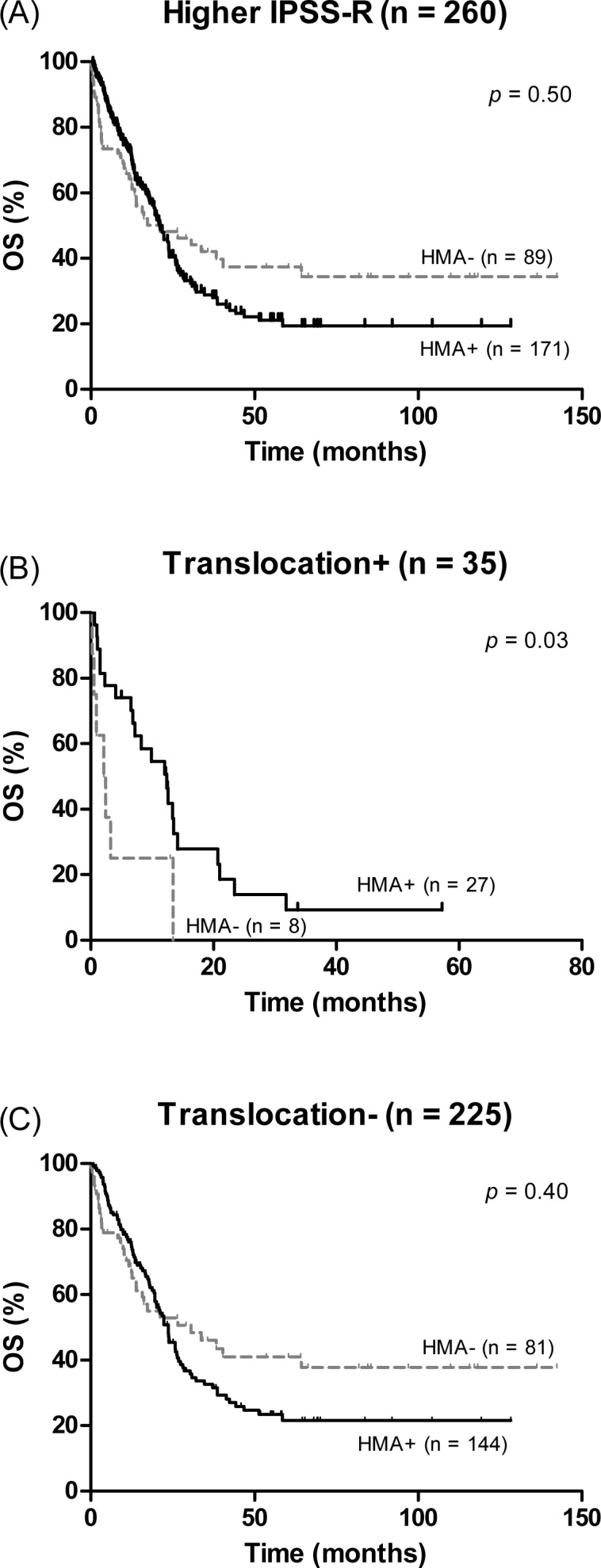
Impact of hypomethylating agents (HMAs) on overall survival (OS) in IPSS-R high and very high risk group. (A) Survival curves in patients treated with HMAs or not treated with HMAs. Impact of HMAs on OS in the subgroup of patients with chromosomal translocation (B) and without chromosomal translocation (C).

Since the proportion of cases with chromosomal translocations increased as the IPSS-R scores increased, we performed a separate analysis to evaluate the prognostic impact of translocation in the different IPSS-R cohorts. Patients with very low, low, and intermediate risk groups were excluded because there were only five patients with translocation in these cohorts; there were 260 patients in the IPSS-R high and very high risk group. Variables including older age at diagnosis, presence of chromosomal translocation, MK, CK, and IPSS-R very high risk group were significantly associated with poorer OS and LFS. Since disease-modifying treatments such as HMAs and HSCT are recommended treatment in the higher risk group, we analysed the effect of these treatments on OS and LFS. Among 260 patients, 40 (15.4%) received HSCT and 171 (65.8%) received HMAs; 121 received azacitidine, and 50 received decitabine, with a median of 4 cycles (range, 1–26). The median OS and LFS were significantly longer in the patients who received HSCT compared to those not receiving HSCT (NR versus 17.4 months, *P* < 0.01 and NR versus 13.5 months, *P* < 0.01, respectively). In contrast, treatment with HMAs was not associated with statistically significant improvement in OS (median 21.0 [18.1–23.9] months for patients treated with HMA versus 21.0 [3.8–38.7] months for the others, *P* = 0.50) (Fig 3A) or LFS (median 17.8 [13.9–21.6] versus 16.2 [7.6–24.8], *P* = 0.35). Also, HMA treatment was not associated with better OS when analysis was restricted to 156 patients with chromosomal abnormality (median OS 21.0 months for both HMA treated patients and the rest, *P* = 0.50). Notably, treatment with HMAs significantly improved OS in patients with translocations (median 2.1 versus 12.4 months, *P* = 0.03) (Fig 3B), whereas this survival difference was not observed for patients without translocation (30.6 months for not treated versus 23.7 months for treated groups, *P* = 0.40) (Fig 3C).

The results from a multivariate analysis of these higher-risk patients, which included age, sex, WHO subtype, MK, CK, translocation, IPSS-R, HMAs, and HSCT indicated that only chromosomal translocation and HSCT were significantly associated with both LFS and OS (Table 4).

**Table 4:** Significant independent predictors of OS and LFS in IPSS-R High and Very High risk patients (n = 260).

Variables	HR for OS (95% CI)	*p*-value	HR for LFS (95% CI)	*P*-value
Translocation				
No	1		1	
Yes	1.72 (1.08–2.73)	0.02	1.65 (1.04–2.60)	0.03
CK				
No	1		1	
Yes	1.77 (1.22–2.57)	< 0.01	1.48 (0.98–2.23)	0.06
HSCT				
No	1		1	
Yes	0.26 (0.15–0.45)	< 0.01	0.23 (0.14–0.40)	<0.01

Abbreviations: HR, hazard ratio; CI, confidence interval; OS, overall survival; IPSS-R, revised International Prognostic Scoring System; CK, complex karyotype; HSCT, hematopoietic stem cell transplantation.

Because HSCT significantly influenced outcome for these high risk patients, we analysed the effect of HMA on prognosis after excluding the 40 patients who had undergone HSCT. The results were virtually the same in the remaining 220 patients; i.e., that treatment with HMA was associated with better outcome only in patients with chromosomal translocation (median OS 2.1 months for patients not treated with HMA versus 12.0 months for patients treated with HMA, *P* = 0.01).

### Analysis of the prognostic factors in patients who received HMAs

We next investigated the association of chromosomal translocation and the clinical outcome of patients treated with HMAs. We excluded 4 patients with an IPSS-R very low risk score. A total of 377 patients with IPSS-R low to very high risk scores had received HMAs; 267 received azacitidine and 110 received decitabine. The median duration of time between diagnosis and HMA initiation was 22 days (range, 1–3128). Among the 377 patients, 27 (7.2%) had a chromosomal translocation.

Response to HMA was evaluable in 271 patients; 22 of these patients had chromosomal translocations. Response was defined as achieving complete remission (CR), complete marrow response (mCR), partial response (PR), and hematologic improvement (HI). Stable disease without HI, and progressive disease (PD) were considered non-response. Response to HMAs were associated with the presence of chromosomal abnormality (response rate [RR] 41.1% for patients with normal karyotype *vs*. 27.4% for those with abnormal karyotype, *P* = 0.048). However, chromosomal translocation was not associated with the RR of HMAs (35.3% for patients without translocation *vs*. 27.3% for those with translocation, *P* = 0.45) (S2 Table).

In univariate analysis, older age, translocation, MK, higher IPSS-R, and not receiving HSCT were correlated with shorter OS. Chromosomal translocation was independently associated with poorer OS (HR 1.71, 95% CI 1.04–2.82, *P* = 0.03) in multivariate analysis (Table 5). Other variables independently associated with OS were MK (HR 1.68, 95% CI 1.09–2.60, *P* = 0.02), IPSS-R (high risk: HR 0.86, 95% CI 0.57–1.31, *P* = 0.49; intermediate risk: HR 0.54, 95% CI 0.34–0.85, *P* < 0.01; low risk: HR 0.22, 95% CI (0.12–0.39), *P* < 0.01), and HSCT (HR 0.43, 95% CI 0.27–0.67, *P* < 0.01). Chromosomal translocation was also correlated with poorer LFS in multivariate analysis with the same variables as entered in the regression model (HR 1.86, 95% CI 1.17–2.95, *P* < 0.01), whereas MK did not remain in the model.

**Table 5:** Significant independent predictors of OS and LFS in patients treated with hypomethylating agents (n = 377).

Variables	HR for OS (95% CI)	*p*-value	HR for LFS (95% CI)	*P*-value
Translocation				
No	1		1	
Yes	1.71 (1.04–2.82)	0.03	1.86 (1.17–2.95)	<0.01
MK				
No	1		-	
Yes	1.68 (1.09–2.60)	0.02	-	-
IPSS-R				
Very High	1		1	
High	0.86 (0.57–1.31)	0.49	0.67 (0.47–0.97)	0.03
Intermediate	0.54 (0.34–0.85)	< 0.01	0.39 (0.27–0.58)	<0.01
Low	0.22 (0.12–0.39)	< 0.01	0.16 (0.10–0.27)	<0.01
HSCT				
No	1		1	
Yes	0.43 (0.27–0.67)	< 0.01	0.37 (0.24–0.57)	<0.01

Abbreviations: HR, hazard ratio; CI, confidence interval; OS, overall survival; IPSS-R, revised International Prognostic Scoring System; CK, complex karyotype; HSCT, hematopoietic stem cell transplantation.

## Discussion

In our study of 751 MDS patients from the Korea MDS Registry, we identified chromosomal translocation in 5.7% of our cohort of patients with MDS. Chromosomal translocation was associated with higher BM blast percentage at diagnosis, in accordance with a higher proportion of RAEB-1 and RAEB-2 subtypes. Translocation was an independent prognostic factor for OS and LFS in the entire patient population, as well as in subgroups of IPSS-R higher risk and HMA-treated populations. Furthermore, in the IPSS-R higher risk group, the survival benefit of HMA was observed only for patients with translocations. To the best of our knowledge, no studies have previously reported these findings.

MDS is cytogenetically very heterogeneous and the incidence of specific aberrations varies considerably, with some being relatively frequent and some very rare. Chromosomal translocation has particularly low frequency, which makes establishing correlations between translocations and patient characteristics and prognoses difficult. Costa et al [7] reported that among 5,654 patients with MDS and CMML, 2,014 (36%) exhibited chromosomal abnormalities including 213 (10%) translocations, which translates into 3.8% of the whole population. In our cohort, the frequency of chromosomal translocations was slightly higher at 5.7% of all patients with MDS, representing 13.7% of patients with an abnormal karyotype. Only a few studies have addressed the prognostic impact of chromosomal translocations. Dvorak et al reported that seven patients with t(2;11)(p21;q23) without an myeloid/lymphoid leukaemia gene rearrangement had a favourable prognosis with a median survival of 72 months [9]. Ai et al reported that patients with non-t(6;9) and non-inv(3) balanced chromosomal rearrangements exhibited short OS similar to patients with IPSS-R poor risk cytogenetics, and that *SRSF2* mutation might be also be associated with worse outcome in these patients [10]. The prognostic impact of chromosomal translocation as a whole, rather than for a specific translocation, has only been evaluated by Nomdedeu et al (11). In this study of 1,653 patients with an abnormal karyotype from the Spanish MDS registry, translocations were identified in 168 patients (10.2%) [11]. Similar to our findings, translocation was associated with high BM blast at diagnosis (7%), RAEB-1 and RAEB-2 (49.9%), and CK (53%), as well as with a significantly shorter survival (median OS, 1.0 year) and a higher incidence of AML transformation (21%, subhazard ratio 1.5, *P* = 0.008), although translocation lost its significance in the multivariate analysis on OS and LFS. They concluded that the poor prognosis of patients with chromosomal translocations was mainly due to a higher incidence of CK in this group. The primary difference between the Nomdedeu et al study and the current analysis is that the former only included patients with abnormal karyotype and included patients with CMML and other MDS/myeloproliferative diseases (10.7%). Here, we have shown consistent results that chromosomal translocation was an independent predictor of adverse outcome in the entire patient population, in the IPSS-R high and very high risk subgroup, and in the subgroup with abnormal karyotype. However, our conclusion needs to be verified in other datasets.

Another notable aspect in our study is that the presence of an MK was not an independent predictor of OS and LFS. The question of whether MK represents an independent prognostic factor of survival has been a focus of attention with many studies devoted to the subject. MK has been reported as a poor prognostic indicator in the whole patient population [17–20], as well as in patients treated with HMAs [21,22]. However, Valcárcel et al reported that the prognostic value of MK is the result of its strong association with a number of chromosomal abnormalities [23]; our study supports this finding by showing that in the context of CK, MK loses its prognostic significance.

In randomized phase III trials, azacitidine demonstrated a survival benefit in higher-risk patients with MDS [24] and decitabine showed superior progression-free survival compared to best supportive care, although a survival advantage was not shown [25,26]. As there are limited treatment options for patients who are not eligible for HSCT, HMAs represent the recommended treatment for high risk patients with MDS [27]. However, whether a survival benefit of HMAs over conventional treatments truly exists and whether IPSS-R is a useful predictor to help guide treatment in the real world MDS population is controversial. In comparison, the OS benefit in patients treated with azacitidine was shown via meta-analysis [28]. However, a recent study in Taiwan reported that a better outcome with azacitidine treatment was only observed for the IPSS-R very high risk group (median OS, 15.2 months vs. 7.5 months) [19]; additionally, in another study of 821 patients with higher-risk MDS in Spain, no significant advantage for azacitidine-treated patients was observed in terms of OS or LFS [29]. The reason for this difference between randomized clinical trials and population based studies is unclear; thus, identification of the biological and clinical variables related to HMA response is of value.

Notably, the survival benefit of HMAs was only observed for patients with chromosomal translocation in the IPSS-R high, very high risk subgroup in our study cohort. To date, only a few studies have focused on the impact of molecular mutation on treatment response to HMAs and these have reported contradictory results. One study found that mutation of *TET2*, a methylation pathway gene, was associated with an increased response to HMAs [30]. Conversely, another study demonstrated that wild type *TET2* and *DNMT3A* were associated with better progression-free survival, whereas mutation of *ASXL2* (a histone-modifying gene) and wild type *SF3B1* (a splicesomal gene) was associated with better OS [31]. Due to the small number of patients with chromosomal translocation, it is hard to draw conclusion. Nevertheless, our findings indicate that the assessment of chromosomal translocation, not all kinds of chromosomal abnormalities, might help identify patients who could derive benefit from HMA treatment, and this finding should be validated in other large cohorts.

The strength of our study is that it involves a large cohort of patients from the Korea MDS Registry and it reflects clinical daily practice. The main characteristics of the enrolled patients including the distribution of cytogenetic risk groups and IPSS-R score are similar to those reported in previous studies, and our study cohort should be representative of this disease. In addition, we have assessed the impact of chromosomal translocation from various angles; i.e., by analysing subgroups with abnormal karyotype, CK, and IPSS-R poor and very poor cytogenetics.

In summary, we demonstrated the clinical significance of chromosomal translocation in MDS. Chromosomal translocation is an independent predictor of adverse outcome and has an additional prognostic value in discriminating patients with MDS and higher risk IPSS-R who might benefit from HMA treatment. Further studies in other populations are needed to validate the clinical relevance of translocation in patients with MDS.

## Supporting Information

S1 Fig Overall survival according to the presence of monosomal karyotype (MK) in (A) the whole population, and (B) patients with complex karyotype (CK), and (C) patients with IPSS-R poor and very poor cytogenetics.
(JPG)

S2 Fig Overall survival and leukemia-free survival according to presence of translocation, excluding 7 patients with t(5q). (JPG)

S1 Table Multivariate analyses for OS and LFS in 744 patients after excluding 7 patients with t(5q). (DOCX)

S2 Table Association of chromosomal abnormality and response to HMAs. (DOCX)
