# Supplementary figures and images for: Prognostic Impact of IPSS-R and Chromosomal Translocations in 751 Korean Patients with Primary Myelodysplastic Syndrome
Source: PLoS One. 2016 Nov 8;11(11):e0166245. doi: 10.1371/journal.pone.0166245 (PMC5100959; doi:10.1371/journal.pone.0166245)

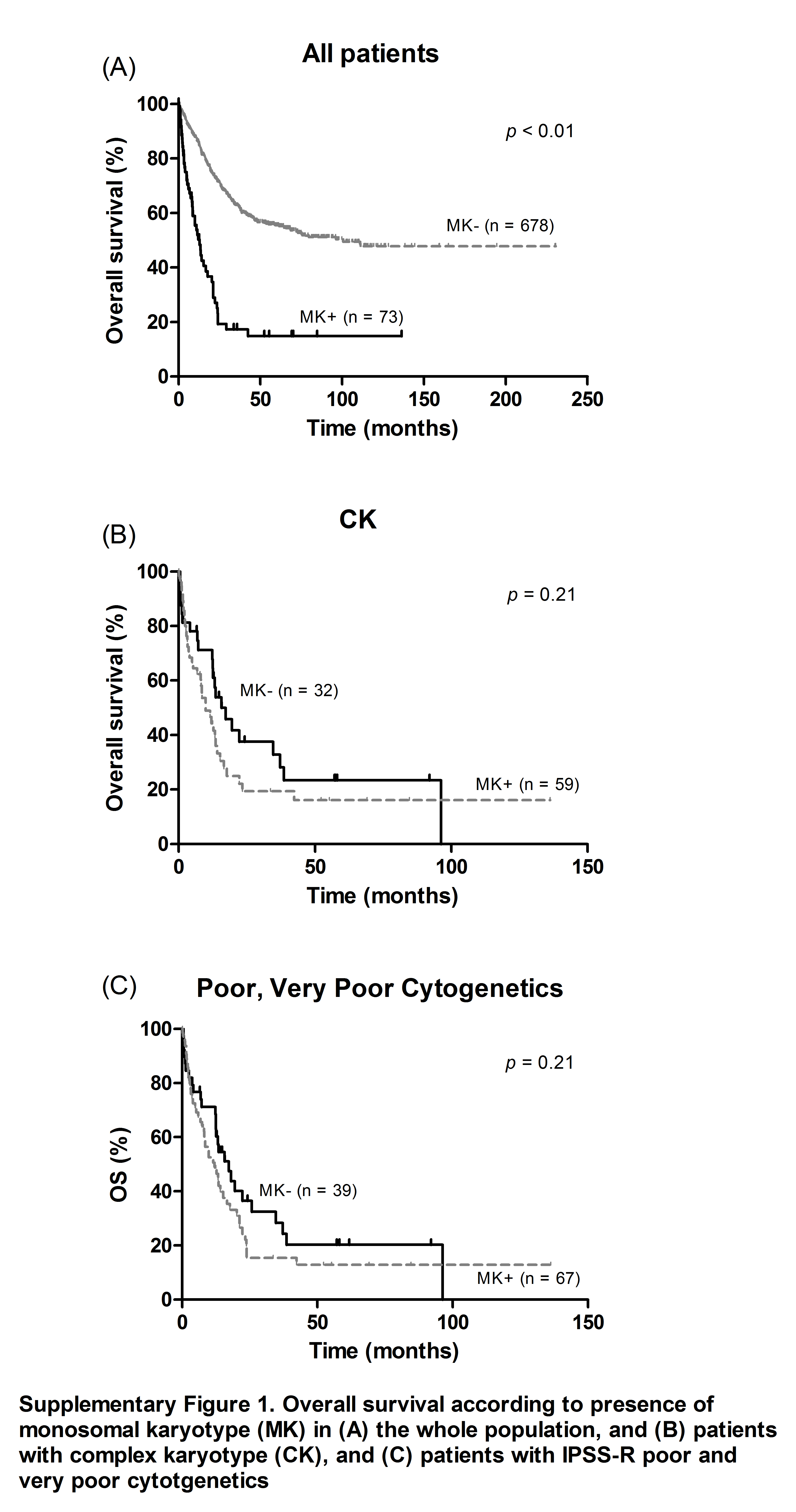

Supplement: S1 Fig — Overall survival according to the presence of monosomal karyotype (MK) in (A) the whole population, and (B) patients with complex karyotype (CK), and (C) patients with IPSS-R poor and very poor cytogenetics. (JPG) [file pone.0166245.s001.jpg]

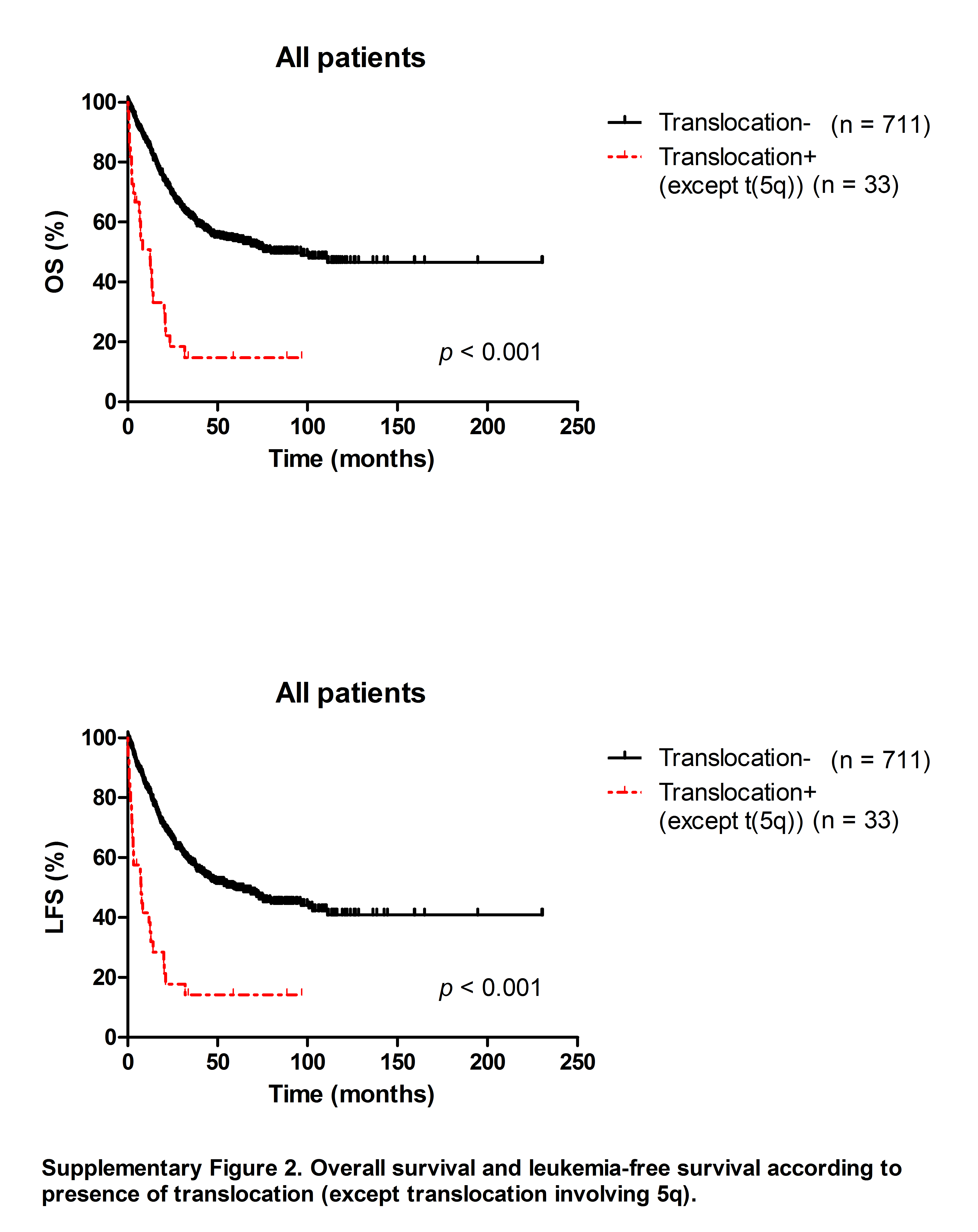

Supplement: S2 Fig — (JPG) [file pone.0166245.s002.jpg]
