# Supplementary material for: Prognostic Impact of IPSS-R and Chromosomal Translocations in 751 Korean Patients with Primary Myelodysplastic Syndrome
Source: PLoS One. 2016 Nov 8;11(11):e0166245. doi: 10.1371/journal.pone.0166245 (PMC5100959; doi:10.1371/journal.pone.0166245)
Supplement: S1 Table — (DOCX) [file pone.0166245.s003.docx]

**Supplementary Table 1. Multivariate analyses for OS and LFS in 744 patients after excluding 7 patients with t(5q)**

| **Variables** | **HR for OS (95% CI)** | ***p*-value** | **HR for LFS (95% CI)** | ***P*-value** |
| --- | --- | --- | --- | --- |
| Age |  |  |  |  |
| <60 | 1 |  | 1 |  |
| ≥60 | 2.10 (1.61 – 2.72) | <0.01 | 2.11 (1.64 – 2.70) | <0.01 |
| Sex |  |  |  |  |
| Female | 1 |  | 1 |  |
| Male | 1.37 (1.06 – 1.75) | 0.02 | 1.24 (0.98 – 1.57) | 0.08 |
| Translocation |  |  |  |  |
| No | 1 |  | 1 |  |
| Yes (Except t(5q)) | 1.89 (1.21 – 2.95) | 0.01 | 1.80 (1.17 – 2.77) | <0.01 |
| CK |  |  |  |  |
| No | 1 |  | 1 |  |
| Yes | 1.79 (1.22 – 2.61) | <0.01 | 1.61 (1.13 – 2.31) | <0.01 |
| IPSS-R |  |  |  |  |
| Very Low | 1 |  | 1 |  |
| Low | 2.39 (0.95 – 5.99) | 0.06 | 2.65 (1.06 – 6.61) | 0.04 |
| Intermediate | 5.18 (2.10 – 12.77) | <0.01 | 5.92 (2.41 – 14.56) | <0.01 |
| High | 8.67 (3.27 – 19.92) | <0.01 | 9.06 (3.68 – 22.33) | <0.01 |
| Very High | 9.42 (3.66 – 24.25) | <0.01 | 13.02 (2.11 – 33.16) | <0.01 |

Abbreviations: HR, hazard ratio; CI, confidence interval; IPSS-R, Revised International Prognostic Scoring System; OS, overall survival; CK, complex karyotype; NSS, not statistically significant.
