# Supplementary material for: Prognostic Impact of IPSS-R and Chromosomal Translocations in 751 Korean Patients with Primary Myelodysplastic Syndrome
Source: PLoS One. 2016 Nov 8;11(11):e0166245. doi: 10.1371/journal.pone.0166245 (PMC5100959; doi:10.1371/journal.pone.0166245)
Supplement: S2 Table — (DOCX) [file pone.0166245.s004.docx]

**Supplementary Table 2. Association of chromosomal abnormality and response to HMAs**

|  | Response  N (%) | Non-response  N (%) | *P*-value |
| --- | --- | --- | --- |
| Chromosomal abnormality |  |  |  |
| No | 60 (41.1) | 86 (58.9) | 0.048 |
| Yes | 34 (27.4) | 90 (72.6) |  |
| Chromosomal translocations |  |  |  |
| No | 88 (35.3) | 161 (64.7) | 0.446 |
| Yes | 6 (27.3) | 16 (72.7) |  |

Abbreviations: HR, hazard ratio; CI, confidence interval; IPSS-R, Revised International Prognostic Scoring System; OS, overall survival; CK, complex karyotype; NSS, not statistically significant.
